# A complicated way of boiling water: nuclear safety in water history

**DOI:** 10.1007/s12685-020-00258-0

**Published:** 2020-11-17

**Authors:** Siegfried Evens

**Affiliations:** KTH Royal Institute of Technology, Stockholm, Sweden

## Abstract

Water and nuclear reactors are much closer intertwined than usually perceived. First, water is the source of the steam that drives the turbines of most nuclear power plants around the world. Next to generating electricity, water is the key to preventing accidents in nuclear plants. As uranium keeps on generating heat when the power plant is turned off, its core needs to be cooled continuously. This crucial connection between water and nuclear is focus of the paper. Nuclear safety will appear as relying heavily on earlier knowledge, institutions, and regulatory frameworks, which were related to water. The three parts of this article discuss technologies, actors and risks of nuclear power. Studying water as a resource in a much broader sense than being boiled for steam shows how determining water is to make nuclear power function. As this paper is part of a special issue, Water History in the time of COVID-19, it has undergone modified peer review.

## Introduction

A nuclear power plant is in fact just a very complicated way of boiling water. This humorous and perhaps slightly cynical statement is a common *boutade* among nuclear engineers. Yet, while it is certainly not an innocent one—it can be used to downplay the clearly major risks and apparent specificities of nuclear power plants—there is some truth in it. Most of the nuclear reactors that provide electricity to our homes today use enormous amounts of water. They are sited next to large water bodies, such as rivers, lakes, and seas, and use this water as a resource. To give an example: one reactor at the Forsmark nuclear power plant in Sweden takes in 50,000–60,000 L of water per second. Furthermore, the nuclear power plant of Forsmark has three reactors, so the entire plant probably takes in around 150,000 L of water per second.^1^

Why does a nuclear power plant need so much water? To generate electricity. When uranium atoms are split in the reactor core, an enormous amount of energy is released. The released energy heats up water, which is turned into steam. The energy from that steam then drives a turbine, which generates electricity. This method is the dominant way of generating nuclear power around the world. They are called light water reactors, because they use normal water as a coolant, and are different from reactors that use heavy water, liquid metal, gas, air, or other substances as a coolant. Another type of light water reactor exists too. In a boiling water reactor (BWR), the water around the core is boiled directly and the steam from that boiling process drives the turbine. It thus only has one cooling circuit, in contrast to the pressurised water reactor (PWR), in which the water is prevented from boiling and the radioactive water from the core is separated from the steam that drives the turbine. That is why it has two circuits, with a steam generator that separates them. In a nuclear power plant, water thus functions as a heat exchanger—a function that water also has in countless other industries. The set-up of a nuclear power plant particularly resembles other thermal power plants, most notably the ones that are fired by coal or oil.

Apart from generating electricity, water has an even more important function in a nuclear power plant: it is the key to preventing accidents. Contrary to any other boiler-based power plant, where a resource such as coal or oil is fired, uranium does not stop generating heat when the power plant is turned off. A chain reaction in the reactor core cannot stop abruptly and will continue to generate large amounts of heat, so the core needs to be cooled continuously. When the cooling flow stops, the core heats up and melts under its own heat. This is called a meltdown and it is widely regarded as the worst possible nuclear accident. The effects from such a meltdown can be a radioactive release into the environment, and it can be caused by a simple pipe break or valve failure.

One would think that this crucial connection between water and nuclear has been studied profoundly, not the least by historians, but the opposite is true. Environmental historians who study nuclear issues, even those who have integrated technological perspectives, have mostly focused on radioactive or thermal pollution in water bodies rather than how those water bodies are used as a resource.^2^ Water historians, on the other hand, have a great deal of experience with studying water as a resource, but nuclear power plants have generally been absent from their radar.^3^ While steam is a recurring theme in history of technology, most water historians tend to stop researching the water from the moment it boils. In this way, many of the industrialisation processes that rely heavily on water stay out of the picture of our historical understanding on water. Conversely, historians such as Joachim Radkau and David Edgerton have already shown that technologies in nuclear power plants are not all high-tech, but we know very little on how this centrality of water and steam has actually affected developments in nuclear safety.^4^

This article presents empirical material to make a conceptual claim. On an empirical level, it is shown that nuclear safety was not a novel scientific, technological, or regulatory domain at its inception in the 1940s and 1950s. Instead, it relied heavily on earlier knowledges, institutions, and regulatory frameworks, which were related to water. This water connection became increasingly important as nuclear power expanded as an energy source and light water reactors became the prevalent reactor type in the world throughout the 1960s, 1970s, and 1980s. This continuity is shown in three different ways over the three parts of this article: (1) the technologies that nuclear power plants use, (2) the actors that managed and regulated them, and (3) the risks that these technologies entailed and how actors attempted to prevent them. The focus is primarily on the United States, France, and Sweden, but international organisations and other countries are also included in the analysis when relevant. The conclusion will assess the conceptual point of this article: looking at water as a resource beyond the boiling point shows how determining water is in the operation and safeguarding of nuclear power, and by extension for many other industries. Nuclear history is thus also a water history, and vice versa.

## Technologies

Water has been inherent in the development of nuclear fission—the partitioning of uranium atoms to produce energy—as a technology. Yet, this should not give the impression that the evolution to water as the dominant coolant for nuclear power was self-evident. After the first Atoms for Peace conference in 1950, water-cooled reactors were used as a technopolitical tool for the promotion of nuclear power. In taking up the leadership in the development of nuclear energy and promoting the technology abroad, the United States and its Atomic Energy Commission (AEC) could ensure that other countries would invest their time and money in that rather than in the production of bombs. However, in order to secure this leadership, it needed a strong domestic civilian nuclear energy program, and water was the key to do that.^5^

An important reason for this to succeed was the military. The installations at Oak Ridge, where the work on the first American atomic bombs had been carried out during World War II, had used light water as a coolant. The technology was later adopted by admiral Hyman Rickover, who used it to develop nuclear submarines.^6^ This led to the design and operation of the first light water reactor: the pressurised water reactor (PWR), which used a primary circuit to cool the core and transport the heat to a second circuit, which propelled the submarine. In 1954, Rickover also began to supervise the construction of the Shippingport plant: the first nuclear power plant for civil purposes, designed as a prototype for attracting investors, which also used the pressurised water design.^7^

Experiences from the military, however, explain only partially the success of water as a coolant. There was an even larger continuity with pre-war industrialisation as well, which made water-cooled reactors a more interesting investment. The pressurised water reactor that Rickover pioneered within the US Navy, relied, in turn, heavily on traditional ways of naval steam propulsion used in steam ships or non-nuclear submarines. It also relied heavily on the advancements in the power industry in the latter half of the 19th and beginning of the twentieth century. In a thermal power plant, coal or oil are burnt in a boiler, which heats up water. The steam generated in the boiler drives a turbine. As we have seen, a light water reactor operates in similar way. Yet, when the first light water reactors were being constructed, engineers encountered the limits of what boilers and pressure vessels could do.^8^ Nuclear power plants were just one step in a much longer process of ever-increasing power plant capacity and thermal efficiency. Larger power plants and more energy production meant more pressure and higher temperatures in the boilers and other pressure equipment, such as valves, steam generators, turbines, and tubes and pipes, thus constantly pushing the limits of technology and safety.^9^ This pushed engineers to improve materials and add more safety devices.

The pressurised water system, which had roots in both the Navy and the power industry, was not the most efficient from an economic perspective, however. Having one circuit and allowing the water to boil—the way in which coal-fired power plants work, for instance—would be more efficient and cheaper. Some engineers even suggested it was safer, since the formation of too much steam would effectively shut down the fission process. After some experiments in the 1950s, such a boiling water reactor (BWR) had also become a credible alternative to the pressurised water reactor.^10^ Still, even this did not convince investors, and aggressive marketing techniques with scientifically questionable quantitative data were needed to really push the water-cooled reactors by what James Jasper calls the “force-feeding of atomic development”. The government’s persistence proved successful, but many nuclear power plants ended up being less efficient and cheap than prognosed.^11^

The rather aggressive promotion of light water reactors with optimistic statistics did not only occur in the US. Following the strategy of Atoms for Peace, light water reactors became an export product *and* a political tool. A cooperation treaty between the United States and Euratom in 1958 foresaw that the US would supply the Euratom member states with enriched uranium (U-235), money for research collaboration, and—most importantly—reactors. Westinghouse, the energy company that sold pressurised water reactors, took part in the agreement in order to export its pressurised water reactors to Europe.^12^ The deal between Euratom and the United States was heavily opposed by the French government and the Commission à l’énergie atomique (CEA). France’s national utility, Electricité de France (EDF), on the other hand, which had much experience in operating large thermal power plants, was interested.^13^ Up to that point, France had only built reactors that use gas as a coolant. Especially the CEA spearheaded this French technology, and EDF played the second violin. In the first French nuclear plants, which were also used for creating plutonium for military purposes, EDF only operated an auxiliary thermal installation for generating power, comprised of a cooling circuit with a turbine.^14^ Later, when EDF began to operate entire installations itself, top officials criticised the design for not being efficient and economically viable enough.^15^ Hence, the deal between the US and Euratom was a tremendous opportunity for EDF to build a Westinghouse pressurised water reactor. Despite acting in direct opposition to the French government and the CEA, the nuclear power plant of Chooz was realised. Located on the border, it was co-operated with the Belgians and one of Europe’s first light water reactors.^16^

Around the same time, Sweden also made the step towards light water reactors. The main state-owned utility, Vattenfall, was enthusiastic, since it shared EDF’s conviction that a nuclear reactor was just an advanced steam engine. Atomenergi AB, Sweden’s atomic agency, saw the nuclear program more as a military and patriotic tool, just as the CEA did. When Sweden finally abandoned its nuclear weapons program in 1960, it opened the door for the light water reactor. Especially ASEA, an industrial company specialised in manufacturing electrical equipment, saw an opportunity for supplying reactors. It was particularly interested in the boiling water reactors from General Electric (GE). Yet, as opposed to the French, who managed to agree on a favourable licensing agreement with Westinghouse, ASEA eventually ended the negotiations with GE and—quite remarkably—designed its own boiling water reactor. In the end, Sweden built 4 nuclear power plants with 12 light water reactors. Many more were planned, but a strong anti-nuclear movement and a referendum ended things abruptly.^17^

Around the middle of the 1960s, however, the AEC lost much of its interest in light water reactors and turned towards fast breeders instead. These are reactors in which the chain reaction is not moderated and coolants such as sodium or molten salt are used. In the words of James Jasper, the AEC had abandoned its very offspring.^18^ This did not stop the promotion of light water reactors abroad, however. After the construction of Chooz, EDF remained particularly open to American reactors. Just as in Sweden, the energy dependency problems of the Oil Crisis in the 1970s opened the way to the massive introduction of water-cooled reactors in France, called the Messmer Plan, with the strategy to go *tout nucléaire*. The succession of Charles De Gaulle by Georges Pompidou increased options for such reactors too. However, the outcome was the result of the *guerre des filières*, a ‘war of the systems’ between the CEA and EDF: gas-graphite against light-water. From that moment on, EDF prioritised nuclear over anything else—something it had not done with the gas-graphite design.^19^ However, this should come as no surprise: for EDF, these light water reactors were just better thermal power plants.

## Actors

Now that it is shown which factors led to the dominance of water as the primary coolant in the nuclear power industry, this part will look closer at the actors that made it possible—several of them were already mentioned: the AEC, Westinghouse, General Electric, the CEA, EDF, Vattenfall, ASEA, or the household names in the nuclear sector. However, there are also many actors involved in the construction, operation, and management of nuclear power plants that are less ‘nuclear’ and also less known, but who play a crucial role as well. Almost all of these have their origins in the study, production, or usage of steam, water, and pressure technologies. Their story is one of continuity, with nuclear just being one of the industrial applications they dealt with.

Perhaps one of the most underestimated and overlooked actors in the nuclear sector is the American Society of Mechanical Engineers (ASME). This engineering association was founded in 1880, largely as an answer to the increasing number of steam boiler and pressure vessel explosions. In 1914, ASME was first in standardizing the construction and operation of boilers and published the Boiler and Pressure Vessel Code. As boilers increased in size, complexity, and pressure capacity, ASME updated the code and included more and more pressure equipment into its regulations, up to the point that the Code covered almost all cooling circuits in water and steam-reliant industries.^20^ As American industrialists were wary of state intervention, or “ramming down regulations down the throats of American citizens”, as they called it, this method of self-regulating was widely supported.^21^ However, even if ASME’s contributions to industrial safety are undeniable, the fact that the Code was written by the same people who had to enforce it, caused continued safety concerns.^22^ As a result, when the first light water reactors were built, large parts of it were subjected to an aged code, which was established half a century earlier by mechanical engineers specialized in thermal hydraulics, not nuclear engineers.

The French model was very different. While the regulation of steam equipment was a private arrangement in the United States, it was a state affair in France, run by the Corps des Mines (CdM). As a state engineering corps traditionally specialised in mechanical engineering, including metallurgy and thermodynamics, it had considerable expertise primarily in pressure vessels. Although not technically part of the state apparatus, the corps and its engineers were tightly intertwined with state affairs, especially within the Ministry of Industry. The Mines began as an engineering association which was delegated some responsibilities in the mining sector by the state. After the Second World War, it widened its operations to all sectors related to pressure technologies, most notably the power industry.^23^ From the middle of the nineteenth century onwards, the corps helped to write steam technology regulations and was tasked with inspecting the compliance with it. Their most important regulatory tool was the pressure limit test (*épreuve*). Before a pressure component left the manufacturer, and after that every 10 years, the component would be tested by a Mines engineer on whether it could hold the allowed pressure or not. When France began to build light water reactors, these pressure tests had to be done on reactor parts too, which made the Corps des Mines an important actor in the construction and operation of nuclear power plants.^24^

The same state-centered approach was apparent in Sweden. The Tryckkärlskommissionen, a part of the Ingenjörsvetenskapsakademien (IVA), elaborated and enforced steam technology regulations. It did this in collaboration—and from the 1960s onwards steered by—a department of the Ministry of Industry, called Arbetsskyddsstyrelsen. Large parts of the cooling circuits thus became subject to old industrial worker’s protection laws.^25^ Yet, on the spectrum between state-centered and privately run, Sweden probably finds itself somewhere in between France and the US. While the connection with the government was clear, the IVA functioned way more as the ASME, as a consortium of different companies, including nuclear ones.

It is in the inspection of nuclear power plants that the institutional continuity related to water technologies is perhaps the clearest. Numerous organizations that were tasked in the 1960s and 1970s with the inspection of nuclear installations had accumulated much experience in inspecting pressure vessels, pipes, or marine technologies. In Sweden, one of the key reactor inspectors, Kiwa Inspecta, has its roots in a Dutch firm that inspected pipes, called Keuringsinstituut voor Waterleidingartikelen, while the steam engineering association Ångpanneföreningen grew to be an influential nuclear consultancy firm.^26^ In the Netherlands, inspections were carried out by the Dienst voor het Stoomwezen (literally translatable as “Steam Service”).^27^ Similarly, the Belgian company Association Vincotte started out as an inspector of boilers delegated by the state before becoming involved in the nuclear sector.^28^ In France, Bureau Veritas, a multinational which has its origins in the inspection and certification of steam ships, went nuclear as well.^29^ Links with steam technologies gave labor inspectorates the prerogative to inspect nuclear power plants in many countries.^30^ Of course, not all this old expertise was immediately relevant for the nuclear reality and there were clear boundaries to this continuity. Still, their expertise with water technologies gave them relevant expertise, which made them well-equipped to take on the important task of inspecting nuclear power plants.

Even the actors that produced and manufactured the reactors often relied on decades of expertise with steam technologies. Babcock and Wilcox, by the middle of the twentieth century a world leading boiler and manufacturer, also constructed many of the nuclear pressure vessels in the US and abroad. The iron and steel mill of Le Creusot, also called Petit Creusot, owned by the old industrial company Schneider et Cie, constructor of countless steam applications, including ships and locomotives, became the main construction site for the manufacturing and assembling of the components of the primary and secondary circuits of the nuclear power plants when France decided to go *tout nucléaire* by constructing large numbers of pressurized water reactors. The location of the iron and steel mill in the region Bourgogne-Franche-Comté rendered the local administration of the Corps des Mines a disproportionately powerful actor in the French nuclear industry.^31^ At Le Creusot, different parts of reactor components were assembled, but all these parts came from manufacturers that were not necessarily experienced with the design, construction, or operation of nuclear power plants, which worried the local Mines inspectors a great deal.^32^ Similarly, multiple pressure vessels for nuclear power plants in the Netherlands, Spain, Belgium, and other countries were all manufactured at the drydock of Rotterdam. Just like Babcock and Wilcox or Schneider, its expertise in metallurgy and steam made this place an ideal site to manufacture reactors.^33^

It would be a mistake to see nuclear power plants as the exclusive playing field of nuclear engineers and physicists. The development, construction, operation, and safeguarding of light water reactors did involve the work of scientists and engineers from many disciplines. Thermal hydraulics and mechanical engineering were crucial to nuclear engineering as a discipline.^34^ Since the launch of the first university programs in nuclear engineering up until today, thermal hydraulics and fluid dynamics have remained core subjects. This traditional core interacted with the rising discipline of nuclear physics, and for nuclear power plants both traditions were indispensable.^35^ Furthermore, the production and operation of the various water circuits in a reactor involve other engineering experts, like material engineers to safeguard the integrity of the steel, or water chemists to make sure that the water is of sufficient quality. The siting of nuclear power plants usually involves hydrologists to analyze the water bodies that might be used as a coolant source. The subsequent modification of that water body necessitates the construction of canals and dykes, hence bringing civil engineers on stage. Apart from the physicists, who are expert in the sole technology that distinguishes a nuclear power plant from other thermal power plants, all other engineers and scientists involved in the nuclear sector have a strong connection with water or steam. It is this connection that is also important when it comes to preventing accidents in nuclear power plants.

## Disasters

If nuclear power plants are just a complicated way of boiling water, are nuclear accidents then only more complicated boiler accidents? Without taking consideration the risk of radioactive fall-out, they actually are: it is widely accepted in the nuclear sector that the worst possible thing that can happen in a reactor is a steam explosion. In a pressurized water reactor, such an explosion would be caused by the formation of steam bubbles in the pressure vessel. That phenomenon would then, in turn, be caused by a loss of coolant accident (LOCA), in which a faulty pipe, tube or valve would disrupt the flow of water to the reactor core, so that the core is not cooled sufficiently. If such an explosion would be able to damage the outer containment of the nuclear power plant, radiation could be released into the environment. Per Högselius calls this a “nuclear drought”, the fear of every nuclear engineer. More than in any other industry, the consequences of a lack of water can be catastrophic.^36^ It is exactly this what lay at the heart of the meltdowns at Chernobyl in 1986 and Fukushima in 2011.^37^ Nuclear history is abundant with accidents that were “nuclear droughts”. The first nuclear accident in history occurred in Nazi-Germany, in an experimental reactor at the University of Leipzig, where a steam explosion occurred.^38^

The United States also faced several explosions during the early years of reactor development at IdahoFalls,^39^ but perhaps its most eye-opening nuclear accident occurred in the Three Miles Island nuclear power plant in Harrisburg in 1979. Four different failures, all of them in the cooling circuits, interacted with each other to form a perfect storm of accidental conditions.^40^ The nuclear core melted partially and some radioactive release had found its way into the environment, but a full-blown catastrophe was prevented. Three Miles Island set the scene for new debates on nuclear safety. Because many nuclear engineers believed that operator errors were the major cause of accidents, much of the attention in nuclear safety was redirected towards the so-called ‘human factors’, focusing on the training of plant operators.^41^ Three Miles Island instigated the inception of the scholarly field of risk research. It inspired Charles Perrow to write *Normal Accidents*, in which he analyses the risks of so-called system accidents that are caused by a combination of failures that would be very normal in ‘traditional’ industries, but cause large, global, and recurring accidents in complex and tight-coupled systems. Seemingly banal components, such as valves, pipes, tubes, pumps etc., are not banal when part of a technological system in which interactive failures lead to catastrophic situations.^42^ Perrow also points to the fact that this complexity comes from hybridity in nuclear systems, being a blend of new fission technologies and old cooling and heat transfer technologies. For sociologist and nuclear engineers alike, Three Miles Island exposed the thermal roots of nuclear power generation. A number of changes in the design and operation of primary and secondary circuits, in addition to more research into thermal hydraulic issues and especially the risks of steam explosions, rendered water and steam more prominent in nuclear safety practices. This also had the result, quite counterintuitively, that water circuits were perceived even stronger as an integral part of the entire nuclear installation than in the early years of nuclear power. From the 1980s onwards, typical thermal hydraulic problems such as material integrity or control systems, have increasingly been treated as nuclear problems.^43^

Norman Rasmussen had assessed that the worst probable accident in a nuclear power plant was a LOCA, like Three Miles Island.^44^ His Rasmussen report (or WASH 1400, 1975), together with Swedish safety studies, led to a growing insight during the 1970s and 1980s that, rather than large pipe breaks, smaller failures—such as breaks in smaller pipes, tubes, or valves—increase the risk for a meltdown, simply because they occur more often.^45^ Around the same time, corrosion became a big safety issue in nuclear power plants around the world, especially ageing ones. Over time, water and radiation damage pipes and tubes to the extent that a LOCA can occur.^46^ Corrosion has been a major problem in every steam application since the nineteenth century, as the many steam explosions caused by corrosion-clogged pipes and tubes in many industries show.^47^ Yet, when the first nuclear power plants began to be operated, how to prevent corrosion or how radiation exacerbates it still seemed unclear.

This innate thermal hydraulic nature of a nuclear accident had an important consequence on the regulations and governance practices for nuclear safety. Nuclear safety codes, legislations, and guidebooks became a hybrid of measures specifically directed towards the risks of radiation on the one hand, and older laws and regulatory traditions for preventing steam explosions on the other hand—as already mentioned above.^48^ In the United States, the Boiler and Pressure Vessel Code (BPVC) began to be applied to nuclear applications as early as the 1940s. However, the larger pressures and temperature differences in nuclear reactors made adjustments necessary as well.^49^ The BPVC was quickly used for Canadian heavy water reactors. Attempts to standardize regulations globally were largely based on the BPVC, given the dominance of the US in the nuclear field.^50^ With the AEC losing most of its enthusiasm for light water reactors and the accompanying nuclear safety initiatives, despite countries such as Sweden and France just finding this enthusiasm, the Advisory Committee on Reactor Safeguards (ACRS) within the AEC continued to express fear for major safety problems or even accidents.^51^ The successor of the AEC, the Nuclear Regulatory Council (NRC) has remained the prime mover in light water reactor safety.^52^

From the 1960s onwards, the BPVC travelled with the export of light water reactors from the United States to other countries, including Sweden for instance.^53^ There is one exception to this process: France. French engineers—especially those within the patriotic circles of the CEA—had been wary and cautious towards the BPVC. They feared that importing it would lead to too much American influence in French industry. France already had firm national legislation on the safety of pressure technologies, as discussed earlier. This did not prevent that the cooling circuits of Chooz were built, under pressure of Westinghouse and Euratom, according to ASME standards. When France embarked on its *tout nucléaire* project in the 1970s and used Westinghouse technology again, the BPVC was “frenchified”. This meant that crucial elements of the safety code, such as maximum allowed pressures, were adapted to the French context. This adaptation was rather pragmatic. Motives for changes included distrust towards the Americans and protection of French industry against foreign competition. Perhaps the most concrete example of this was the copying of ASME’s four categories of states of a reactor: (1) normal conditions, (2) deviations from normal conditions, (3) emergency conditions, and (4) faulted conditions.^54^ The French copied the classification, but removed the fourth (worst) category, fearing that even recognising the possibility of such a category would lead to increased protests from anti-nuclear movements.^55^ Hence, the French nuclear safety regulations were not only hybrids between radiation safeguards and steam regulations, but also between American and French safety traditions.

## Conclusion

This article has shown in which ways the histories of water management and technology and the ascent of nuclear power generation during the post-war period are intrinsically related. Water was an important factor in the choice for a suitable reactor design, because its presence in the navy and numerous industries rendered it an efficient solution to roll out large reactor parks quickly and cheaply. However, this was only possible due to the actors who drove this process—actors who all had considerable experience and a long history of engaging with water and steam applications. Many of these actors engaged in nuclear safety. Even more than in other thermal power plants, water is the key to preventing deadly accidents, rendering thermal hydraulic expertise essential for nuclear power. Many of the codes and regulations are a hybrid of specific measures for radiation and older industrial safety regulations for preventing steam explosions, just as the plants that they govern are a hybrid of older steam technologies and newer fission technologies.

With this empirical evidence, this article has clearly demonstrated the importance of nuclear technologies for the study of water history. In many ways, nuclear safety has been, and still is, about understanding the behaviour of water, and translating that into codes and practices. Nuclear actors relied heavily on pre-existing industrial knowledge, especially related to thermal hydraulics, and used this knowledge to promote nuclear power and safeguard reactors. This also entails that water history should not only be about water itself, but also about the steam when the water is boiled. Nuclear water studies reveal important continuities and processes of technological change, however old they may be. In fact, this paper has a limited temporal scope, and much research can be done on how these reactor technologies stretch back even farther into time—back to the medieval ages and antiquity. Water has been manipulated by humans and directed into circuits, however complex they may be, for the purpose of power generation for millennia. In this water history, nuclear power is also a part. The history of nuclear power is long—longer than often assumed—and water is the cause of that.

## Footnotes

1 “Vid Forsmark 1 finns det två stora inloppskanaler för kylvatten…”, *Vattenfall*, Facebookpost. 9 May 2019. Union of Concerned Scientists (2019), ‘Omfattade revisionsarbete på Forsmark 3’, Vattenfall, accessed 28 September 2020, https://group.vattenfall.com/se/nyheter-och-press/nyheter-pressmeddelanden/nyheter/ringhals-och-forsmark-arkiv-2013-2016/2014/omfattade-revisionsarbete-pa-forsmark-3.

2 For an overview on perspectives in nuclear history, see Kalmbach (2017).

3 A notable exception is the work of Sara Pritchard, who has written a history on the nuclearisation of the Rhône river in France. This case helped her to theorise the concept’envirotechnical regime’. She has also applied this perspective to the Fukushima accident, which highlighted the important role that water played during the accident. See Pritchard (2011) and Pritchard (2012).

4 Radkau (2008, pp. 314–16) and Edgerton (2008).

5 Krige (2006).

6 Hewlett et al. (1989, p. 191) and Mahaffey (2011, pp. 94–101).

7 Krige (2006, p. 164), Mahaffey (2015, p. 114) and Walker (1992, pp. 18–36).

8 Hewlett et al. (1989, p. 192).

9 Cross (1990, pp. 83–85, 103–4, 151–53, 178), Somerscales (1990) and Major (1990).

10 Hewlett et al. (1989, pp. 191, 255).

11 Jasper (1990, pp. 42–45).

12 ”US-Euratom Legislation signed by President”, *Bulletin from the European Community* 31, Archive of European Integration, Pittsburgh, 1–4: https://aei.pitt.edu/43606/1/A7414.pdf. Mangeon (n.d., p. 136), Foasso (2003) and Krige (2008).

13 Hecht (2009).

14 Hecht (2009, pp. 67–69).

15 Hecht (2009, pp. 95–96).

16 Torres (1996).

17 Kaijser (2020, pp. 244–247), Högselius and Kaijser (2007, pp. 30–33), Fridlund (1999, pp. 194–95) and Glete (1983).

18 Jasper (1990, pp. 51–54).

19 Hecht (2009, pp. 271–323), Jasper (1990, p. 89) and Mangeon (n.d., pp. 133–47).

20 Cross (1990, pp. 25–54).

21 Cross (1990, p. 68).

22 Cross (1990, pp. 67–68).

23 Erhard Friedberg and Dominique Desjeux,”Fonction de l’Etat et rôle des grands-corps: le cas du Corps des Mines” (1972), 19950069/1, Archives Nationales, Paris, 2–8.

24 Mangeon (n.d., p. 140). “Décret du 2 avril 1926 portant règlement sur les appareils à vapeur autres que ceux placés à bord des bateaux” (Paris, Ministry of Public Works, 1926), Legifrance: https://www.legifrance.gouv.fr/affichTexte.do?cidTexte=JORFTEXT000000300042&dateTexte=20160718

25 ”Normernas framtida uppläggning, innehåll och distribution” (9 May 1985), F5t:54 Tryckkärlskommissionen, Centrum för Näringslivshistoria, Stockholm. Short List of Codes, Standards, and Regulations on Boilers and Pressure Vessels” (1962), SC/523-1: Nuclear Power and Reactors—Reactor Design and Construction—Boiler and Pressure Vessel Codes as applied to Reactor Vessels, IAEA Archives, Vienna.

26 Historia (2020), Om (2020), G. Edling, “Letter to Skjöldebrand” (16 December 1985), SC/523-1: Nuclear Power and Reactors—Reactor Design and Construction—Boiler and Pressure Vessel Codes as applied to Reactor Vessels, IAEA Archives, Vienna.

27 J. Kneppelhout, “Letter to the IAEA” (20 August 1963), SC/523-1: Nuclear Power and Reactors—Reactor Design and Construction—Boiler and Pressure Vessel Codes as applied to Reactor Vessels, IAEA Archives, Vienna.

28 R. Skjöldebrand,”The Application of Pressure Vessel Codes to Nuclear Reactor Systems” (s.d.), SC/523-1: Nuclear Power and Reactors—Reactor Design and Construction—Boiler and Pressure Vessel Codes as applied to Reactor Vessels, IAEA Archives, Vienna, 4 & attachment.

29 ‘Nucléaire’, Bureau Veritas France (2020), H. de Senneville, “Letter to the Minister of Industry” (28 January 1967), 19771473/113, Archives Nationales, Paris.

30 "Short List of Codes, Standards, and Regulations on Boilers and Pressure Vessels” (1962), SC/523-1: Nuclear Power and Reactors—Reactor Design and Construction—Boiler and Pressure Vessel Codes as applied to Reactor Vessels, IAEA Archives, Vienna.

31 ”Note contrôle administratif des installations nucléaires” (29 September 1975), 19840190/20, Archives Nationales, Paris, 3–7.

32 ”Influence du rythme de construction sur la qualité des études et de la réalisation” (29 April 1977), 19840190/20, Archives Nationales, Paris. Michel Herblay, “L’Atome de tous les jours: Premier bilan des résultats et des faiblesses de l’industrie nucléaire française”, *L’Expansion* (February 1979), 20050139/110 vol I, Archives Nationales, Paris, 105.

33 S. Havel,”Report on a Meeting of ISO on Reactor Pressure Vessels (5–7 July 1967), O/340-40—Organizations—International Organization for Standardization Vol. II Part IV, IAEA Archives, Vienna.

34 D’Auria (2017), For a history of the field of thermal hydraulics and thermodynamics, see Lemons (2019).

35 See for instance”Civilingenjörsutbildningen på atomenergiområdet: Betänkande med förslag av särskilt tillkallade sakkunniga” (1959), KTH Biblioteket, Stockholm.

36 Högselius (2014), Högselius (2013).

37 The literature on both accidents is vast, but some key works, including official reports on the accident, that highlight the importance of cooling in the accidents are Pritchard (2012), Samuels (2013), Brown (2019); International Nuclear Safety Advisory Group (1986), Medvedev (1992), International Atomic Energy Agency, *The Fukushima Daiichi Accident. Report by the Director General*, 2015, https://inis.iaea.org/Search/search.aspx?orig_q=RN:46110848.

38 Mahaffey (2015, pp. 184–89).

39 Mahaffey (2015 , pp. 345, 405–13).

40 Perrow (2011, pp. 15–32).

41 Pershagen (1989, pp. 388–97), Walker et al. (2010, pp. 57, 58). SC/645 US 1: Three Miles Island Vol IV and SC/563-1: Nuclear Power Plant Control and Instrumentation—Meeting on Procedures and Systems for Assisting an Operator During Normal And Anomalous Nuclear Power Plant Operations; Munich, Germany, 1979-12-05/07; Vol. XIX, IAEA Archives, Vienna.

42 Perrow (2011, pp. 3–14) and Le Coze (1984–2014).

43 Pershagen (1989, pp. 392–93); United States. (1979), United States Congress (2015). NRC, “Fact Sheet: The Accident at Three Miles Island” (1993), https://www.hsdl.org/?view&did=778074. SC/563-1: Nuclear Power Plant Control and Instrumentation—Meeting on Procedures and Systems for Assisting an Operator During Normal And Anomalous Nuclear Power Plant Operations; Munich, Germany, 1979-12-05/07; Vol. XIX, IAEA Archives, Vienna. E. Steele, “Letter to H,J, Laue” (19 July 1979), IAEA Archives, Vienna. “International Experience in the Implementation of the Lessons Learned from the Three Miles Island Incident” (1983), IAEA-TECDOC-294, https://inis.iaea.org/collection/NCLCollectionStore/_Public/14/806/14806222.pdf.

44 Rasmussen et al. 1975 Reactor safety study. An assessment of accident risks in U. S. commercial nuclear power plants. Executive Summary. WASH-1400 (NUREG-75/014). Rockville, MD, USA: Federal Government of the United States, U.S. Nuclear Regulatory Commission.

45 Pershagen (1989, pp. 224–27, 238), D’Auria (2017). Reactor Safety Study: An Assessment of Accident Risks in US Commercial Nuclear Power Plants (USNRC 1975).

46 U.S. Nuclear Regulatory Commission (1984). For France, see”Compte rendu du Section Permanent Nucléaire” (23 June 1978), 19840190/20, Archives Nationales, Paris, 3–6.”Matériaux utilisés dans la construction du circuit primaire principal des chaudières du Contrat Programme” (8 May 1979), 19840190/20, Archives Nationales, Paris. Michel Herblay, “L’Atome de tous les jours: Premier bilan des résultats et des faiblesses de l’industrie nucléaire française”, *L’Expansion* (February 1979), 20050139/110 vol I, Archives Nationales, Paris, 105. “Compte rendu succint de la section permanente nucléaire (23 June 1978), 19910334/20, Archives Nationales, Paris, 1.

47 For the United States, see for instance Cross (1990, pp. 23–35). For France, see for instance 19910335/10 and 19910335/21, Archives Nationales, Paris.

48 For an overview on radiation safety regulations and practices, see Boudia (2007).

49 Cross (1990, pp. 98–114).

50 SC/523-1: Nuclear Power and Reactors—Reactor Design and Construction—Boiler and Pressure Vessel Codes as applied to Reactor Vessels, IAEA Archives, Vienna.

51 Jasper (1990, pp. 51–54).

52 Wilson (2013).

53 Cross(1990, p. 87).”Short List of Codes, Standards, and Regulations on Boilers and Pressure Vessels” (1962), SC/523–1: Nuclear Power and Reactors—Reactor Design and Construction—Boiler and Pressure Vessel Codes as applied to Reactor Vessels, IAEA Archives, Vienna.

54 U. S. Nuclear Regulatory Commission (1973, pp. 1.48–4).

55 ”Compte rendu du cinquième séance” (28 June 1971), 19840190/20, Archives Nationales, Paris, 4–5.
